# Association between sleep quality and urolithiasis among general population in Western China: a cross-sectional study

**DOI:** 10.1186/s12889-022-14187-5

**Published:** 2022-09-20

**Authors:** Sheng Wang, Xianghong Zhou, Shi Qiu, Boyu Cai, Yifan Li, Chichen Zhang, Kunjie Wang, Lu Yang, Lei Chen

**Affiliations:** 1 The Department of Urology, West China Hospital, Department of Sichuan University, West China Hospital of Sichuan University, No. 37 Guoxue Xiang, Chengdu, 610041 Sichuan China; 2 The Department of Neurology, West China Hospital, Department of Sichuan University, West China Hospital of Sichuan University, No. 37 Guoxue Xiang, Chengdu, 610041 Sichuan China

**Keywords:** Sleep quality, Urolithiasis, Pittsburgh sleep quality index, Chinese

## Abstract

**Background:**

Growing number of studies have evidently shown that sleep disorders are associated with the recently increased risk of various diseases in general human population. However, the relationship between sleep quality and urolithiasis condition in humans is still unclear. The present study explored the relationship between quality of sleep and urolithiasis in Chinese population of population, western China and hence investigated the effects of sleep quality on urolithiasis disease.

**Methods:**

A cross-sectional analysis was performed using data from the West China Natural Population Cohort Study (WCNPCS). The data was collected between May 2019 and June 2021. This study evaluated the association between the sleep quality and urolithiasis. The sleep quality was assessed using the Chinese version of the Pittsburgh Sleep Quality Index (PSQI) whereas urolithiasis, as the outcome was a binary variable. Multivariable logistic regression models that adjust the sociodemographic characteristics and health-related factors were used to assess the association between sleep quality and urolithiasis. Interaction was tested in prespecified subgroup of interest.

**Results:**

After adjusting a series of confounding variables, the Pittsburgh Sleep Quality Index scores were found to have a significant positive correlation with the prevalence of urolithiasis (OR: 1.178; 95% CI = 1.083–1.282; *p* < 0.001). The risk of urolithiasis was significantly increased with an elevation of the component Pittsburgh Sleep Quality Index score in sleep latency, sleep duration, habitual sleep efficiency, and daytime dysfunction.

**Conclusions:**

It was evident that there is an association between sleep quality and prevalence of renal stones in natural population in western China regions. Poor sleep quality is related to urolithiasis. The findings of the current study hence highlighted the need for future public health guidelines to develop detailed strategies for improving sleep quality.

**Supplementary Information:**

The online version contains supplementary material available at 10.1186/s12889-022-14187-5.

## Introduction

Urolithiasis is an ancient disease which causes heavy disease and economic burden to patients and their families. However, there is still no specified pathogenesis of kidney stones [1]. Urolithiasis is a global disease, and the incidence and occurrence of urolithiasis are on the rise. Some studies have shown that nephrolithiasis is a highly prevalent disease in the world. Its incidence of is between 7 and 13% in North America, between 5 and 9% in Europe, as well as between 1 and 5% in Asia [2–4]. The disease also has a high recurrence rate, which undoubtedly poses a considerable burden on global public health. The incidence of urolithiasis significantly varies with geography, climate, diet, liquid intake, heredity, sex, occupation, and age [5]. A study conducted by Sorokin et al. [5] compared the International Consultation on Urological Diseases (ICUD) consensus document published in 2008 with the latest recommendations of ICUD2008–2014 and the American Urological Association (AUA) guidelines on various aspects of the relationship between urolithiasis and diet. The researchers, Sorokin et al.*,* concluded that calcium, oxalate, animal protein, carbohydrates, and sodium remains the core dietary risk factors of urinary stone disease [5, 6]. Therefore, it is crucial to identify the potentially modifiable risk factors of the disease. This help to improve the design of prevention programs and reduce the prevalence of urolithiasis.

Currently, sleep is considered to be a key factor in maintaining good health. Epidemiological studies have shown that sleep disorders [7] and mental disorders [8],usually attributed to work stress and environmental deterioration are on the rise around the world [9]. Sleep restriction can lead to both metabolic and endocrine impairment [10, 11]. Elsewhere, studies have shown that sleep posture may lead to unilateral kidney hyperperfusion, which promotes the formation of renal stones [12–14]. However, the previous studies only assessed the correlation between stone laterality and sleep posture hence, the correlation of sleep quality and urolithiasis has not been studied. Therefore, this study aimed to use clustered logistic regression to analyze the potential associations between sleep quality (measured using the Pittsburgh Sleep Quality Index) and urolithiasis as well as to identify the independent contribution of sleep quality to occurrence of urolithiasis in the Chinese population.

## Methods

### Data source and study population

The current research was a cross-sectional analysis based on the data of the West China Natural Population Cohort Study (WCNPCS) obtained between May 2019 and June 2021. The data was collected from the three most populous regions of Sichuan Province (Mianzhu, Longquan, and Pidu) in Western China. The WCNPCS cohort was established in 2019–2021 with a total of 36,075 participants from West China. The WCNPCS cohort was established in 2019–2021 with a total of 36,075 participants from West China. The subjects of WCNPCS are selected from the permanent adult residents of the cooperative community by sequential cluster sampling. The trained full-time staff will carry out face-to-face questionnaire survey, physical examination, biological sample collection and special examination. WCNPCS aims to establish a large-scale forward-looking natural population cohort.

**Fig. 1:**
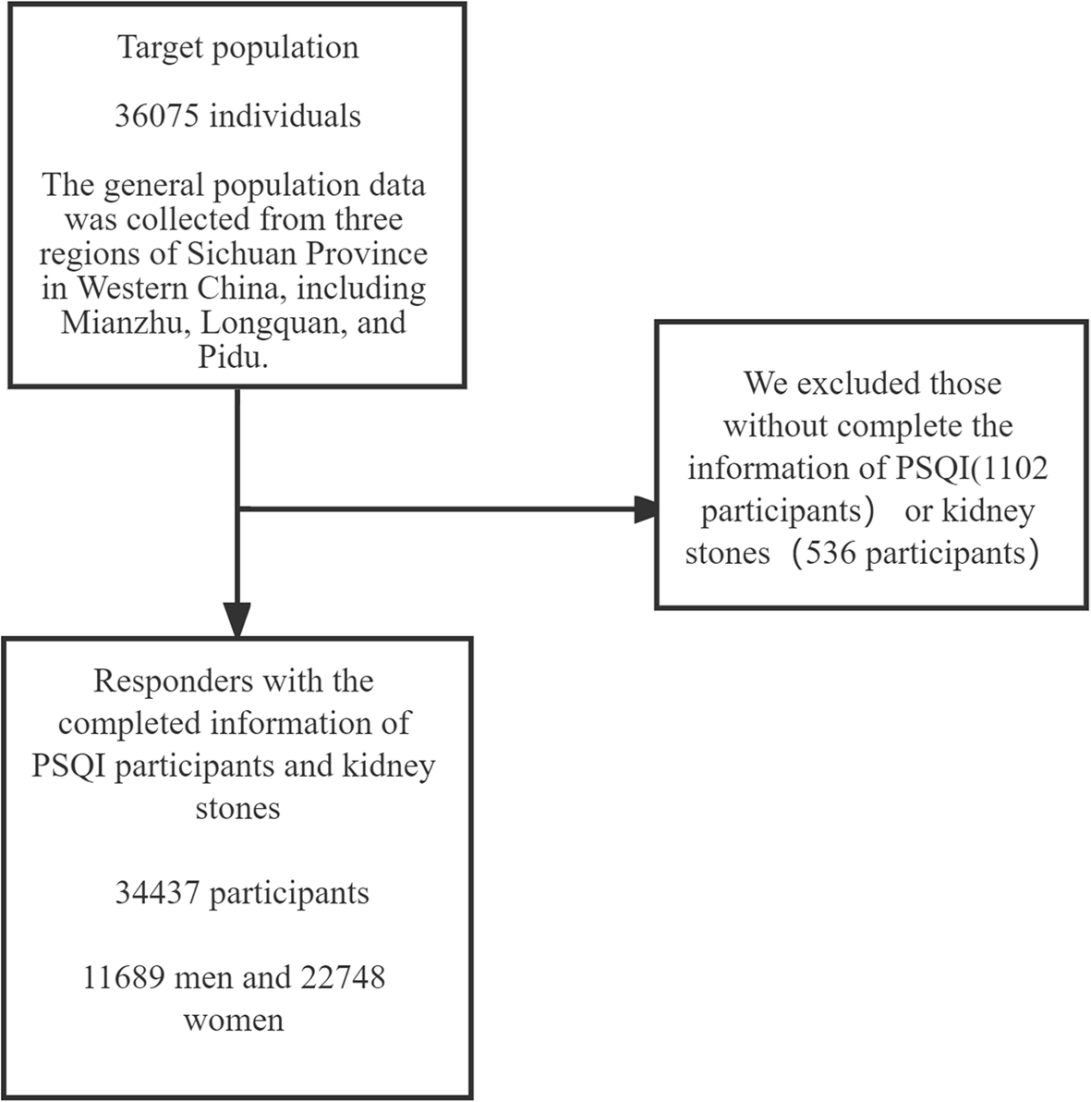
Flow chart of cross-sectional study for prevalence of urolithiasis among adults in West China, 2019–2021

Participants were recruited into the present study on a voluntary basis. Each of them obtained and signed an informed consent before the survey was undertaken. Face-to-face interviews and collection of bio-specimens including serum and urine was conducted by trained medical staff. The specimens were analyzed in the medical laboratory according to the standard experimental procedures (Fig. 1).

A questionnaire survey of 36,075 people was conducted and those without complete information of PSQI (1102 participants) or kidney stones (536 participants) were finally excluded from this study. Therefore, a total of 34,437 participants were lastly included in the study.

All the applied study protocols were in accordance with the Declaration of Helsinki of 1975 with applicable revisions at the time of the present investigation. Approval of the used study protocols was provided by the ethical committee of West China Hospital of Sichuan University and the study was registered with the Chinese Clinical Trial Registry (registration No. ChiCTR1900024623) on July 19, 2019.

### Exposure assessment

Translated PSQI was used to assess the sleep quality. The PSQI is a standard self-reporting questionnaire consisting of 19 items designed to collect the subjective nature of a person’s sleep habits within a month [15]. The PSQI quadruple scale for each entry ranges from 0 to 3. Furthermore, PSQI has been used in several cases to diagnose sleep disorders and proved to have good reliability and validity [16–18].

The translated PSQI assesses different aspects of sleep and reflects seven aspects of sleep problems. The aspects include subjective sleep quality, sleep latency, sleep duration, habitual sleep efficiency, sleep disturbance, use of sleep medication, and daytime dysfunction [15]. The sum of the assessed sleep aspects constitutes a global sleep quality score ranging from 0 to 21 and the higher the score, the lower the sleep quality.

The global PSQI score is higher than 7 and hence can distinguish between people with poor sleep quality and those who sleep well. In Chinese population, the score has high diagnostic sensitivity and specificity of 98.3 and 90.2%, respectively [19].

### Variables

In WCNPCS study, survey participants who answered yes to the question, “Have you ever had a kidney stone?” were considered to have a history of urolithiasis. Potential covariates were identified a priori based on literature review. Continuous variables included in the present study consisted of age, BMI, waist to hip ratio (WHR), Patient Health Questionnaire-9 (PHQ9) score, Generalized Anxiety Disorder (GAD-7) score, and Serum creatinine (Cr).

On the other hand, categorical variables included in this study were: gender (male/female), marital status, education, smoking history, alcohol intake, coffee intake, and tea intake. Smoking is defined as smoking more than 100 cigarettes in a lifetime and drinking is defined as more than 30 g per week in the last 12 months. This definition has been verified in some previous studies [20]. Comorbid conditions of the current study included 1) Diabetes mellitus (DM), 2) congestive heart failure, 3) chronic obstructive pulmonary disease (emphysema and/or chronic bronchitis), 4) coronary artery disease, 5) cancer, and6) hypertension. The total number the reported conditions of the participants, in exception of diabetes, which was separately assessed, was later combined to create an ordinal comorbidity index [21].

### Statistical analysis

The assessed characteristics of the participants were described as mean ± standard deviations (mean ± SD), median (interquartile range) or percentage counts. Chi-square analyses were used to examine the differences of the characteristics among the participants with or without a history of urolithiasis. Multivariable logistic regression analyses were utilized to examine the odds ratios (ORs) and 95% confidence intervals (CIs) for the risk factors of urolithiasis. Urolithiasis status was used as a dependent variable, whereas the sleep quality (Global PSQI score ≤ 7 or > 7) and the seven components of the PSQI (subjective sleep quality, sleep latency, sleep duration, habitual sleep efficiency, sleep disturbance, use of sleep medication, and daytime dysfunction) were used as independent variables.

The ORs and 95% CIs were calculated whereby ORs were adjusted for age in a minimally-adjusted model (Model 1). The ORs were further adjusted for age, BMI, education, marital status, Smoking, drinking, coffee, tea, WHR, PHQ9, GAD7, comorbidity index, DM, physical activity, and Cr in a fully-adjusted model (Model 2). Subgroup analyses were performed and stratified by gender. As metabolic syndrome has a certain influence on the occurrence of stones, we also carried out a corresponding stratified analysis of metabolic syndrome. The *P* values for interaction were tested using the likelihood ratio test. The statistical software packages R (http://www.R-project.org, The R Foundation) and EmpowerStats (http://www.empowerstats.com, X&Y Solutions, Inc., Boston, MA) were used for described statistical analyses. Statistical significant difference was set at *P*-value < 0.05.

## Results

### Participant characteristics

A total of 34,437 participants who meet the inclusion and exclusion criteria were recruited into this study. The various characteristics of the participant are as presented in Table 1 according to urolithiasis history. The average age of the participants with no renal stones was 56.155 ± 12.156 years (SD). The average age of participants with renal stones was 56.156 ± 10.954 years (SD), and 12.2% of the participants were found with urolithiasis.

**Table 1:** Prevalence of urolithiasis by characteristics

Characteristics		Urolithiasis history		Standardize diff.	*P*-value*
	No，*N* = 30,233(M ± SD)/N(%)		Yes, *N* = 4204(M ± SD)/N(%)		
Age	56.155 ± 12.156		56.156 ± 10.954	0.000 (−0.032, 0.033)	0.142
BMI	24.387 ± 3.289		24.852 ± 3.228	0.143 (0.110, 0.175)	**< 0.001**
WHR	0.857 ± 0.065		0.869 ± 0.064	0.188 (0.155, 0.221)	**< 0.001**
PHQ9	1.007 ± 2.235		1.109 ± 2.387	0.044 (0.012, 0.076)	**0.002**
GAD7	1.016 ± 2.430		1.145 ± 2.615	0.051 (0.019, 0.083)	**0.002**
Cr	59.846 ± 20.048		63.061 ± 17.604	0.170 (0.138, 0.203)	**< 0.001**
**Gender**				0.297 (0.265, 0.330)	**< 0.001**
Female	20,504 (67.820%)		2244 (53.378%)		
Male	9729 (32.180%)		1960 (46.622%)		
**Education**				0.046 (0.011, 0.082)	0.209
Primary	9228 (37.289%)		1241 (35.826%)		
Junior	9450 (38.186%)		1377 (39.752%)		
High	3711 (14.996%)		522 (15.069%)		
College	2308 (9.326%)		321 (9.267%)		
Graduate	50 (0.202%)		3 (0.087%)		
**Marital status**				0.092 (0.060, 0.125)	**< 0.001**
Married	26,804 (89.293%)		3800 (91.040%)		
Unmarried	420 (1.399%)		31 (0.743%)		
Divorced	752 (2.505%)		101 (2.420%)		
Widowed	766 (2.552%)		68 (1.629%)		
Separation	1276 (4.251%)		174 (4.169%)		
**Smoking**				0.192 (0.160, 0.225)	**< 0.001**
Current	4519 (14.964%)		866 (20.634%)		
Occasionally	375 (1.242%)		76 (1.811%)		
Nerver	23,951 (79.311%)		2982 (71.051%)		
Ever	1354 (4.484%)		273 (6.505%)		
**Drinking**				0.075 (0.043, 0.107)	**< 0.001**
Yes	10,418 (34.500%)		1555 (37.050%)		
No	18,793 (62.235%)		2470 (58.852%)		
Ever	986 (3.265%)		172 (4.098%)		
**Coffee intake**				0.033 (0.001, 0.065)	0.299
No	27,844 (92.269%)		3905 (92.976%)		
1-2timesperweek	1942 (6.435%)		250 (5.952%)		
3-5timesperweek	199 (0.659%)		26 (0.619%)		
>5timesperweek	192 (0.636%)		19 (0.452%)		
**Tea intake**				0.064 (0.032, 0.096)	**0.001**
No	17,758 (58.848%)		2356 (56.095%)		
1-2timesperweek	4668 (15.469%)		654 (15.571%)		
3-5timesperweek	1533 (5.080%)		247 (5.881%)		
>5timesperweek	6217 (20.602%)		943 (22.452%)		
**Comorbidity index**				0.110 (0.078, 0.143)	**< 0.001**
0	16,878 (55.882%)		2127 (50.595%)		
1	12,501 (41.390%)		1933 (45.980%)		
2	804 (2.662%)		137 (3.259%)		
> = 3	20 (0.066%)		7 (0.167%)		
**DM**				0.060 (0.028, 0.092)	**< 0.001**
No	27,865 (92.476%)		3810 (90.822%)		
Yes	1868 (6.199%)		316 (7.533%)		
Prediabetes	399 (1.324%)		69 (1.645%)		
**Physical activity**				0.044 (0.012, 0.076)	**0.028**
Inactive	10,012 (33.155%)		1467 (34.962%)		
Notsuffcient	4094 (13.557%)		524 (12.488%)		
Suffcient	16,092 (53.288%)		2205 (52.550%)		

Mean and SD for continuous variables, % for Categorical variables

*P*-value*: Kruskal-Wallis test or chi -square test

The bold values considered statistically significant

Most participants were married (88.9%), female (66.1%), and had completed junior school or higher education (69.6%). The patients with urolithiasis history were found to have higher BMI, waist to hip ratio (WHR), Patient Health Questionnaire-9 (PHQ9) score (Assess the patient’s tendency to be depressed), and Generalized Anxiety Disorder (GAD-7) score, Serum creatinine (Cr) as compared with the participants without history of urolithiasis. Therefore, it was evident that there were significant differences in gender, marital status, smoking, drinking, tea drinking, comorbidity index, glucose metabolism, and physical activity among the participants of the present study (*P* < 0.05).

### Multivariate regression analysis

To explore the association of sleep quality with urolithiasis, it was observed that the total PSQI scores of the participants with a history of urolithiasis were significantly higher than those without the history. Results of the current study showed that the risk of urolithiasis was significantly increased with an elevated component PSQI score in sleep latency, sleep duration, habitual sleep efficiency, sleep disturbance and daytime dysfunction. In addition, it was found that there was the decrease in component PSQI score in the subjective sleep quality (*P* < 0.05; Table 2). Results of logistic regression analyses on the associations between the PSQI scores and the prevalence of urolithiasis in participants of the present study were as shown in Table 3. Results from the crude model showed that the prevalence of urolithiasis was positively associated with the PSQI scores. Further, the total PSQI scores were still observed to have a significant positive correlation with the prevalence of urolithiasis after adjustments were made for the confounding variables (OR: 1.178; 95% (CI) = 1.083–1.282; *p* < 0.001). In detail, as shown in Table 3, the risk of urolithiasis was significantly increased with the elevated component PSQI score in Sleep latency (OR: 1.217; 95% (CI) =1.082–1.368; *P* < 0.01), Sleep duration (OR: 1.219; 95% (CI) =1.056–1.408; *P* < 0.01), Habitual sleep efficiency (OR: 1.181; 95% (CI) =1.066–1.309; *P* < 0.01) and Daytime dysfunction (OR: 1.446; 95% (CI) =1.186–1.763; *P* < 0.001).

**Table 2:** Comparison between urolithiasis and non-urolithiasis groups

Variable	Urolithiasis		Standardize diff.	***P***-value*
	No, ***N*** = 30,233，N(%)	Yes, ***N*** = 4204, N(%)		
PSQI grouping			0.073 (0.041, 0.106)	< 0.001
<=7	21,530 (71.214%)	2852 (67.840%)		
> 7	8703 (28.786%)	1352 (32.160%)		
Subjective sleep quality			0.073 (0.041, 0.106)	< 0.001
0	5205 (17.216%)	697 (16.579%)		
1	18,076 (59.789%)	2410 (57.326%)		
2	5771 (19.088%)	922 (21.931%)		
3	1181 (3.906%)	175 (4.163%)		
Sleep latency			0.072 (0.040, 0.104)	< 0.001
0	7679 (25.399%)	1013 (24.096%)		
1	6830 (22.591%)	875 (20.814%)		
2	10,181 (33.675%)	1445 (34.372%)		
3	5543 (18.334%)	871 (20.718%)		
Sleep duration			0.060 (0.028, 0.093)	0.004
0	9219 (30.493%)	1182 (28.116%)		
1	14,503 (47.971%)	2043 (48.597%)		
2	3900 (12.900%)	570 (13.559%)		
3	2611 (8.636%)	409 (9.729%)		
Habitual sleep efficiency			0.060 (0.027, 0.092)	0.004
0	13,855 (45.827%)	1840 (43.768%)		
1	6617 (21.887%)	896 (21.313%)		
2	3895 (12.883%)	561 (13.344%)		
3	5866 (19.403%)	907 (21.575%)		
Sleep disturbance			0.098 (0.066, 0.131)	< 0.001
0	4127 (13.651%)	479 (11.394%)		
1	22,971 (75.980%)	3182 (75.690%)		
2	3041 (10.059%)	526 (12.512%)		
3	94 (0.311%)	17 (0.404%)		
Use of sleep medication			0.011 (−0.021, 0.043)	0.938
0	29,224 (96.663%)	4067 (96.741%)		
1	334 (1.105%)	46 (1.094%)		
2	251 (0.830%)	31 (0.737%)		
3	424 (1.402%)	60 (1.427%)		
Daytime dysfunction			0.106 (0.074, 0.138)	< 0.001
0	23,076 (76.327%)	3027 (72.003%)		
1	4289 (14.186%)	679 (16.151%)		
2	2081 (6.883%)	340 (8.088%)		
3	787 (2.603%)	158 (3.758%)		

% for Categorical variables: *P*-value* was calculated by chi -square test

**Table 3:** Cluster logistic regression models explaining urolithiasis by variables in the seven sleep quality domains and global PSQI score

PSQI components	Crude，***N*** = 34,437，OR (95%CI) ***P*** value	P for trend	Adjust model 1^**a**^, ***N*** = 33,796，OR (95%CI) ***P*** value	P for trend	Adjust model 2^**b**^, ***N*** = 26,556, OR (95%CI) ***P*** value	P for trend
Global PSQI score (≤ 7)	1		1		1	
>7	1.173 (1.094, 1.257) < 0.00001		1.171 (1.091, 1.257) 0.00001		1.178 (1.083, 1.282) 0.00015	
Subjective sleep quality		0.00071		0.00139		0.01259
0	1		1		1	
1	0.996 (0.910, 1.089) 0.92400		0.995 (0.909, 1.089) 0.90635		0.974 (0.879, 1.080) 0.62140	
2	1.193 (1.074, 1.326) 0.00101		1.192 (1.072, 1.326) 0.00120		1.200 (1.060, 1.359) 0.00390	
3	1.107 (0.927, 1.321) 0.26312		1.081 (0.902, 1.295) 0.39852		1.023 (0.823, 1.270) 0.83944	
Sleep latency		0.00011		0.00012		0.00021
0	1		1		1	
1	0.971 (0.882, 1.069) 0.55056		0.976 (0.886, 1.075) 0.62658		0.959 (0.854, 1.078) 0.48454	
2	1.076 (0.988, 1.172) 0.09393		1.077 (0.987, 1.174) 0.09423		1.092 (0.986, 1.210) 0.09151	
3	1.191 (1.081, 1.312) 0.00040		1.194 (1.083, 1.317) 0.00038		1.217 (1.082, 1.368) 0.00102	
Sleep duration		0.00034		0.00028		0.00341
0	1		1		1	
1	1.099 (1.018, 1.186) 0.01553		1.098 (1.016, 1.185) 0.01774		1.110 (1.017, 1.212) 0.01926	
2	1.140 (1.025, 1.268) 0.01617		1.149 (1.030, 1.281) 0.01257		1.132 (0.999, 1.283) 0.05160	
3	1.222 (1.083, 1.378) 0.00113		1.230 (1.087, 1.392) 0.00101		1.219 (1.056, 1.408) 0.00676	
Habitual sleep efficiency		0.00036		0.00021		0.00122
0	1		1		1	
1	1.020 (0.936, 1.110) 0.65441		1.026 (0.941, 1.118) 0.56319		1.032 (0.936, 1.137) 0.53197	
2	1.085 (0.980, 1.200) 0.11526		1.095 (0.988, 1.213) 0.08431		1.096 (0.974, 1.232) 0.12879	
3	1.164 (1.069, 1.268) 0.00047		1.177 (1.078, 1.286) 0.00028		1.181 (1.066, 1.309) 0.00146	
Sleep disturbance		< 0.00001		< 0.00001		0.00208
0	1		1		1	
1	1.193 (1.078, 1.321) 0.00064		1.190 (1.074, 1.319) 0.00091		1.144 (1.021, 1.282) 0.02069	
2	1.490 (1.306, 1.701) < 0.00001		1.484 (1.297, 1.697) < 0.00001		1.262 (1.079, 1.476) 0.00366	
3	1.558 (0.922, 2.634) 0.09786		1.572 (0.929, 2.661) 0.09178		1.506 (0.846, 2.681) 0.16394	
Use of sleep medication						
0	1		1		1	
1	0.990 (0.726, 1.349) 0.94749		1.025 (0.751, 1.398) 0.87811		1.078 (0.760, 1.530) 0.67334	
2	0.887 (0.610, 1.291) 0.53217		0.923 (0.634, 1.343) 0.67392		0.952 (0.623, 1.455) 0.82077	
3	1.017 (0.774, 1.335) 0.90435		1.037 (0.789, 1.363) 0.79489		1.132 (0.835, 1.533) 0.42445	
Daytime dysfunction		< 0.00001		< 0.00001		< 0.00001
0	1		1		1	
1	1.207 (1.104, 1.320) 0.00004		1.209 (1.104, 1.324) 0.00004		1.175 (1.057, 1.306) 0.00288	
2	1.246 (1.104, 1.405) 0.00037		1.239 (1.096, 1.401) 0.00060		1.193 (1.039, 1.371) 0.01257	
3	1.530 (1.285, 1.823) < 0.00001		1.498 (1.254, 1.790) < 0.00001		1.446 (1.186, 1.763) 0.00027	

Outcome: Stones

Crude: no covariates were adjusted

Model 1^a^: adjusted for age

Model 2^b^: adjusted for Age; BMI; Education; Marital status; Smoking; Drinking; Coffee; Tea; WHR; PHQ9; GAD7; comorbidity index; DM; Physical activity; Cr

The outcomes of subgroup analyses which explored the relationship between PSQI score and urolithiasis stratified by metabolic syndrome of the participants were as shown in Supplementary Tables 2&3. the relationship between sleep and urolithiasis in this population (OR: 1.181; 95% CI: 0.964–1.448; *P* = 0.10804) is not as significant as that in non-metabolic syndrome participants (OR: 1.174; 95% CI: 1.069–1.288; *P* < 0.001). The outcomes of subgroup analyses which explored the relationship between PSQI score and urolithiasis stratified by gender of the participants were as shown in Table 4. It was found that there was a higher correlation between PSQI score and urolithiasis among female participants (OR: 1.043; 95% CI: 1.027–1.059; *P* < 0.001) as compared with that of male participants (OR: 1.029; 95% CI: 1.010–1.049; *P* = 0.0034). Moreover, it was observed that there was a significant interaction between sleep quality and gender (*P* for interaction = 0.0354).

**Table 4:** Logistic regression models explaining urolithiasis by variables in gender

	Crude, OR (95%CI) ***P*** value	Adjust model 1^**a**^, OR (95%CI) ***P*** value	Adjust model 2^**b**^, OR (95%CI) ***P*** value
**Female**			
PSQI scores (for each additional point)	1.054 (1.041, 1.066) < 0.0001	1.052 (1.040, 1.065) < 0.0001	1.043 (1.027, 1.059) < 0.0001
**Male**			
PSQI scores (for each additional point)	1.025 (1.009, 1.040) 0.0017	1.029 (1.013, 1.045) 0.0003	1.029 (1.010, 1.049) 0.0034
**P for interaction**	0.0045	0.0051	0.0354

Outcome: stones

Crude: no covariates were adjusted

Model 1^a^: adjusted for age

Model 2^b^: adjusted for Age; BMI; Education; Marital status; Smoking; Drinking; Coffee; Tea; WHR; PHQ9; GAD7; comorbidity index; DM; Physical activity; Cr

## Discussion

The purpose of this study was to explore the relationship between sleep quality and urolithiasis. In addition, the current study also evaluated the effect of sleep quality on urolithiasis among general population in Western China. Results of the analysis showed that poor sleep quality is significantly related to urolithiasis. Further, it was evident that the incidence of urolithiasis was positively correlated with sleep disorder score and negatively correlated with subjective sleep quality score.

Patients with urolithiasis showed more significant sleep latency than that of the patients without urolithiasis. This means that the limited sleep time in patients with urolithiasis will be compressed, creating the possibility of a vicious circle. Consequently, limitation in sleep time has an impairment on cognitive function, metabolic, and endocrine [10, 11, 22]. In animals, genetic disruption of circadian rhythms produces characteristics of chronic kidney disease that includes elevated serum creatinine, glomerular and tubular damage, and cortical fibrosis [23]. However, it has been found that only a few longitudinal analyses of individuals without chronic kidney disease have studied the relationship between sleep time and changes in renal parameters in human studies. The studies showed that the shorter the amount of sleep, the faster the annualized decline in EGFR and the greater the risk of a 30% decline in EGFR after 11 years [24–26].

Recent research shows that there is a significant correlation between shorter sleep time and rapid renal function decline, which has nothing to do with many identified risk factors for CKD [26]. This may explain the role of sleep duration and habitual sleep efficiency in formation of urolithiasis. However, there is still need for further studies to clarify the underlying mechanism of the decline in sleep and renal functions. As shown in Table 3, it was evident that there was a positive association between the daytime dysfunction and formation of urolithiasis.

Previous studies have shown that poor sleep quality may be associated with drowsiness and fatigue, that may indirectly cause reduced physical activities during the day [27, 28]. Further, according to a cross-sectional survey carried out in southern China, it was noted that exercise is a protective factor for formation of renal stones (OR: 0.840; 95% CI: 0.808–0.973; *P* < 0.05) [29] and a decrease in physical activities is a reasonable factor for the poor sleep quality to cause the formation of urolithiasis.

The underlying mechanism of the correlation between poor sleep quality and formation of urolithiasis may also be caused by the effect of a decrease in sleep quality on physiological changes such as metabolism, hormone secretion, and appetite regulation [30], whereby the effect may in turn cause the formation of urolithiasis. For example, it has been found that the secretion of Ghrelin and Leptin as well as the response of neurons to food stimuli are affected by sleep restriction and appetite regulation, thus affecting both the choices of food and calorie intake [10, 31]. However, according to International Consultation on Urological Diseases (ICUD) Consensus Document, 2014 and AUA guidelines [6], limitation of refined carbohydrates intake to less than 20 g per day was recommend to prevent renal stone formation. In addition, other studies have shown that the quality of sleep can affect obesity [30, 32], which may also be a risk factor for the formation of urolithiasis [5].

Overall, the changes in dietary intake such as limited intake of refined carbohydrates, exercise, and metabolism caused by sleep may play a key role in the formation of urolithiasis. The relationship between sleep quality and urolithiasis is not fully understood. Therefore, there is need for future longitudinal or follow-up studies and to confirm the mechanisms of the possible correlation.

In addition, results of the subgroup analyses in the present study also found an interesting phenomenon that the effect of sleep quality on the prevalence of urolithiasis in women was greater than that in men. The results also showed that women have a higher incidence of renal stones among the participants in this study. It has been generally shown that the prevalence and incidence of kidney stones have been dominated by men for more than a century [33] but there is some evidence that the gender gap (GR) is currently narrowing [5].

Between 1970 and 2000 it was found that the GR rose from 3:1 to 1.3 in the United States with the ratio of women growing at an annual rate of 1.9% and the ratio of men annually declining by 1.7% for the 30 years [34]. Although men have a higher incidence of renal stones in the overall population, it was evident that women in the group with poor sleep quality were more significantly affected by the stones as compared with men. This could be related to the effect of sleep quality on estrogen secretion in women.

This study recommends that there is need for further exploration to confirm the underlying mechanism of the possible correlation between sleep quality indifferent gender and formation of urolithiasis. However, it can be explained that women face multiple pressure factors from work, family, economy, and society as compared with men. Therefore, the existence of sleep disorders in women population may be caused by stress and sleep disorders may also cause stress [30]. It is evident that this vicious circle will significantly affect the health of women in the world. Therefore, good sleep quality is an essential factor to overall human health, including protecting women from the formation of urolithiasis.

This study had some limitations. First, although to the best of our knowledge, this study was the first to use general population data to analyze the relationship between sleep quality and prevalence of kidney stones in Chinese population, the causal relationship between sleep quality and urolithiasis may not be clarified because of the applied cross-sectional study design. Furthermore, longitudinal and intervention studies may provide a better understanding of the causal relationship. Second, although we have measured creatinine as a representative of renal functions, measurement of sleep posture, anatomic alterations, different hormones, or laboratory indicators such as sex hormones, Vitamin D, and eGFR, were not conducted yet they could be an intermediary between sleep quality and urolithiasis. Finally, some variables such as smoking, drinking alcohol, drinking tea, drinking coffee, and exercise may have had some reporting or recall biases.

## Conclusion

It is evident that there is a correlation between sleep quality and prevalence of urolithiasis in natural population in western China. Poor sleep quality (Subjective sleep quality, Sleep latency, Sleep duration, Habitual sleep efficiency, Sleep disturbance, and Daytime dysfunction) is related to urolithiasis. The findings of this study showed the need for future public health guidelines to develop detailed strategies for improvement of sleep quality. The strategies may include preventing and intervening risk factors that affects sleep quality as well as recommending the best sleep time to effectively reduce the incidences of urolithiasis.

## Supplementary Information

**Additional file 1: Supplementary Table1.** Reported Kidney Stone Prevalence by Country and Year.

**Additional file 2: Supplementary Table 2.** Cluster logistic regression models explaining urolithiasis by variables in the global PSQI score.

**Additional file 3: Supplementary Table 3.** Logistic regression models explaining urolithiasis by variables in Metabolic syndrome.

## Data Availability

All data is available from the corresponding author on reasonable request.

## Abbreviations

PSQI: Pittsburgh Sleep Quality Index
BMI: Body Mass Index
WHR: Waist to hip ratio
PHQ9: Patient Health Questionnaire-9
Cr: Serum creatinine
SD: Standard deviations
EGFR: Estimated glomerular filtration rate
