# Supplementary material for: Association between sleep quality and urolithiasis among general population in Western China: a cross-sectional study
Source: BMC Public Health. 2022 Sep 20;22:1787. doi: 10.1186/s12889-022-14187-5 (PMC9490950; doi:10.1186/s12889-022-14187-5)
Supplement: Supplementary file 1 — Additional file 1: Supplementary Table1. Reported Kidney Stone Prevalence by Country and Year. [file 12889_2022_14187_MOESM1_ESM.docx]

Supplementary table1: Reported Kidney Stone Prevalence by Country and Year

| Country | Year | region | Prevalence | gender | |
| --- | --- | --- | --- | --- | --- |
|  |  |  |  | male | female |
| China | 2019-2020 | west region | 12.2% | 16.7% | 9.8% |
| United States | 2007-2010(1) | nationwide | 8.8% (95% CI, 8.1-9.5). | 10.6% (95% CI, 9.4-11.9) | 7.1% (95% CI, 6.4-7.8) |
|  | 2017–2018(2) | nationwide | NA | 11.9% (95%CI, 9.3-14.5) | 9.4% (95%CI, 7.3-11.4) |
| South Korea | 1998(3) | nationwide | 3.50% | 6.00% | 1.80% |
|  | 2002-2013(4) | nationwide | 11.50% | 12.90% | 9.80% |
| Saudi Arabia | 2017(5) | western region | 6.00% | 6.60% | 5.80% |

1. Scales CD, Jr., Smith AC, Hanley JM, Saigal CS. Prevalence of kidney stones in the United States. European urology. 2012;62(1):160-5.

2. Abufaraj M, Xu T, Cao C, Waldhoer T, Seitz C, D'Andrea D, et al. Prevalence and Trends in Kidney Stone Among Adults in the USA: Analyses of National Health and Nutrition Examination Survey 2007-2018 Data. European urology focus. 2021;7(6):1468-75.

3. Kim H, Jo MK, Kwak C, Park SK, Yoo KY, Kang D, et al. Prevalence and epidemiologic characteristics of urolithiasis in Seoul, Korea. Urology. 2002;59(4):517-21.

4. Tae BS, Balpukov U, Cho SY, Jeong CW. Eleven-year Cumulative Incidence and Estimated Lifetime Prevalence of Urolithiasis in Korea: a National Health Insurance Service-National Sample Cohort Based Study. Journal of Korean medical science. 2018;33(2):e13.

5. Nassir AM. Prevalence and characterization of urolithiasis in the Western region of Saudi Arabia. Urology annals. 2019;11(4):347-52.
