# Supplementary material for: Association between sleep quality and urolithiasis among general population in Western China: a cross-sectional study
Source: BMC Public Health. 2022 Sep 20;22:1787. doi: 10.1186/s12889-022-14187-5 (PMC9490950; doi:10.1186/s12889-022-14187-5)
Supplement: Supplementary file 2 — Additional file 2: Supplementary Table 2. Cluster logistic regression models explaining urolithiasis by variables in the global PSQI score. [file 12889_2022_14187_MOESM2_ESM.docx]

Supplementary Table 2. Cluster logistic regression models explaining urolithiasis by variables in the global PSQI score

| **PSQI components** | **Crude，N=34437，OR (95%CI) P value** |  | **Adjust model 1^a^, N=33796，OR (95%CI) P value** |  | **Adjust model 2^b^, N=26637, OR (95%CI) P value** |  |
| --- | --- | --- | --- | --- | --- | --- |
| Global PSQI score (≤ 7) | 1 |  | 1 |  | 1 |  |
| ＞7 | 1.173 (1.094, 1.257) <0.00001 |  | 1.171 (1.091, 1.257) 0.00001 |  | 1.173 (1.078, 1.276) 0.00021 |  |

Outcome: Stones

Crude: no covariates were adjusted.

Model 1a: adjusted for age

Model 2b: adjusted for Age; BMI; Education; Marital status; Smoking; Drinking; Coffee; Tea; PHQ9; GAD7; comorbidity index; Physical activity; Cr; Metabolic syndrome
