# Supplementary material for: Association between sleep quality and urolithiasis among general population in Western China: a cross-sectional study
Source: BMC Public Health. 2022 Sep 20;22:1787. doi: 10.1186/s12889-022-14187-5 (PMC9490950; doi:10.1186/s12889-022-14187-5)
Supplement: Supplementary file 3 — Additional file 3: Supplementary Table 3. Logistic regression models explaining urolithiasis by variables in Metabolic syndrome. [file 12889_2022_14187_MOESM3_ESM.docx]

Supplementary Table 3. Logistic regression models explaining urolithiasis by variables in Metabolic syndrome

| **PSQI** | Non-Metabolic syndrome  OR (95%CI) P value | Metabolic syndrome  OR (95%CI) P value |
| --- | --- | --- |
| Non-adjusted |  |  |
| Global PSQI score (≤ 7) | 1 | 1 |
| ＞7 | 1.197 (1.107, 1.294) <0.00001 | 1.032 (0.870, 1.224) 0.72038 |
| Adjust I |  |  |
| Global PSQI score (≤ 7) | 1 | 1 |
| ＞7 | 1.189 (1.099, 1.287) 0.00002 | 1.103 (0.928, 1.312) 0.26612 |
| Adjust II |  |  |
| Global PSQI score (≤ 7) | 1 | 1 |
| ＞7 | 1.174 (1.069, 1.288) 0.00077 | 1.181 (0.964, 1.448) 0.10804 |

Outcome: Stones

Crude: no covariates were adjusted.

Model 1a: adjusted for age

Model 2b: adjusted for Age; BMI; Education; Marital status; Smoking; Drinking; Coffee; Tea; PHQ9; GAD7; comorbidity index; Physical activity; Cr; Metabolic syndrome
